# A Determination of Metallothionein in Larvae of Freshwater Midges (*Chironomus riparius*) Using Brdicka Reaction

**DOI:** 10.3390/s8074081

**Published:** 2008-07-10

**Authors:** Ivo Fabrik, Zuzana Ruferova, Klara Hilscherova, Vojtech Adam, Libuse Trnkova, Rene Kizek

**Affiliations:** 1 Department of Chemistry and Biochemistry, and Mendel University of Agriculture and Forestry, Zemedelska 1, CZ-613 00 Brno, Czech Republic; 2 Research Centre for Environmental Chemistry and Ecotoxicology, and Masaryk University, Kotlarska 2, CZ-611 37 Brno, Czech Republic; 3 Department of Animal Nutrition and Forage Production, Faculty of Agronomy, Mendel University of Agriculture and Forestry, Zemedelska 1, CZ-613 00 Brno, Czech Republic; 4 Department of Chemistry, Faculty of Science, Masaryk University, Kotlarska 2, CZ-611 37 Brno, Czech Republic

**Keywords:** Catalytic Hydrogen Evolution, Brdicka Reaction, Differential Pulse Voltammetry, Environmental Marker, Thiols, Metallothionein, Heavy Metal Contamination

## Abstract

Among wide spectrum of biomolecules induced by various stress factors low molecular mass protein called metallothionein (MT) is suitable for assessment of the heavy metal environmental pollution. The aim of this work was to determine the metallothionein and total thiols content in larvae of freshwater midges (*Chironomus riparius*) sampled from laboratory exposure to cadmium(II) ions and from field studies using differential pulse voltammetry Brdicka reaction. Unique electrochemical instrument, stationary electrochemical analyser Autolab coupled with autosampler, was utilized for the analysis of the samples. The detection limit for MT was evaluated as 5 nM. The larvae exposed to two doses (50 ng/g or 50 μg/g) of cadmium(II) ions for fifteen days under laboratory controlled conditions were at the end of the exposure killed, homogenized and analysed. MT content in control samples was 1.2 μM, in larvae exposed to 50 ng Cd/g it was 2.0 μM and in larvae exposed to 50 μg Cd/g 2.9 μM. Moreover at field study chironomid larvae as well as sediment samples have been collected from eight field sites with different levels of pollution by heavy. The metals content (chromium, nickel, copper, zinc, arsenic, molybdenum, cadmium, tin and lead) in the sediment and or MT content in the chironomid larvae were determined by inductively coupled plasma mass spectrometry or Brdicka reaction, respectively.

## 1. Introduction

The on-line monitoring of a specific pollutant can be performed for only a short time interval, thus the alternative methods for long-term monitoring of pollution are developing. Various species of plants and animals, which are sensitive to higher levels of pollutants or which can synthesize easily detectable biomolecules as a response to environmental pollution, have been used to assess the effects of long-term environmental stress [1-8]. Among wide spectrum of biomolecules induced by various stress factors low molecular mass thiols are suitable for assessment of the environmental pollution because of their main physiological functions in scavenging of reactive oxygen species and detoxification of toxic organic and inorganic molecules via binding with free –SH groups [9]. Metallothioneins (MT) as a group of intracellular, low molecular mass, free of aromatic amino acids and rich in cysteine proteins with molecular weight from 6 to 10 kDa can be considered members of forementioned thiols biomarkers [10-14]. These proteins are abundant through whole animal kingdom, and they were also found in higher plants, eukaryotic microorganisms and some prokaryotes. MT can be found mostly in liver, kidney, pancreas and intestines at animal species. Moreover, MT is accumulated in lysosomes and was found also in nuclei [15].

It has been shown that the MT level increases, when an organism is affected by heavy metals ions. This event can be used for monitoring of environmental contamination by heavy metals [16-18]. Besides stress factors MT level strongly depends on animal specie, analysed tissue, age of an animal, eating habits and likely on others, not yet fully understood and identified factors.

Various analytical techniques and methods can be used to determine MT [11,16,18-41]. One of the most sensitive techniques called Brdicka reaction belongs to wide group of electrochemical methods and measures the hydrogen evolution catalyzed by a protein containing free –SH groups in the presence of Co(III) complex. This method was discovered by Brdicka in 1933 [42-44]. Since the discovery Brdicka reaction has been utilized for determination of MT levels in various animal species (fish, mussels, gastropods) [45-50]. A modification of this method to improve its sensitivity and selectivity has been recently proposed [10,51].

The aim of this work was to determine the metallothionein and total thiols content in larvae of freshwater midges (*Chironomus riparius*) using Brdicka reaction.

## 2. Material and Methods

### 2.1 Chemicals and instruments

Rabbit liver MT (MW 7143), containing 5.9 % Cd and 0.5 % Zn, was purchased from Sigma Aldrich (St. Louis, USA). Tris(2-carboxyethyl)phosphine (TCEP) is produced by Molecular Probes (Evgen, Oregon, USA). Co(NH_3_)_6_Cl_3_ and other used chemicals were purchased from Sigma Aldrich in ACS purity unless noted otherwise. The stock standard solutions of MT at 10 μg/ml was prepared with ACS water (Sigma-Aldrich, USA), reduced by adding of 1 mM TCEP [52] and stored in the dark at − 20 °C. Working standard solutions were prepared daily by dilution of the stock solutions. Deionised water underwent demineralization by reverse osmosis using the instruments Aqua Osmotic 02 (Aqua Osmotic, Tisnov, Czech Republic) and then it was subsequently purified using Millipore RG (Millipore Corp., USA, 18 M′Ω) – MiliQ water. The pH value was measured using WTW inoLab Level 3 with terminal Level 3 (Weilheim, Germany), controlled by personal computer program (MultiLab Pilot; Weilheim). The pH-electrode (SenTix-H) was calibrated by set of WTW buffers (Weilheim).

### 2.2 Larvae of freshwater midges

**Figure 1.**
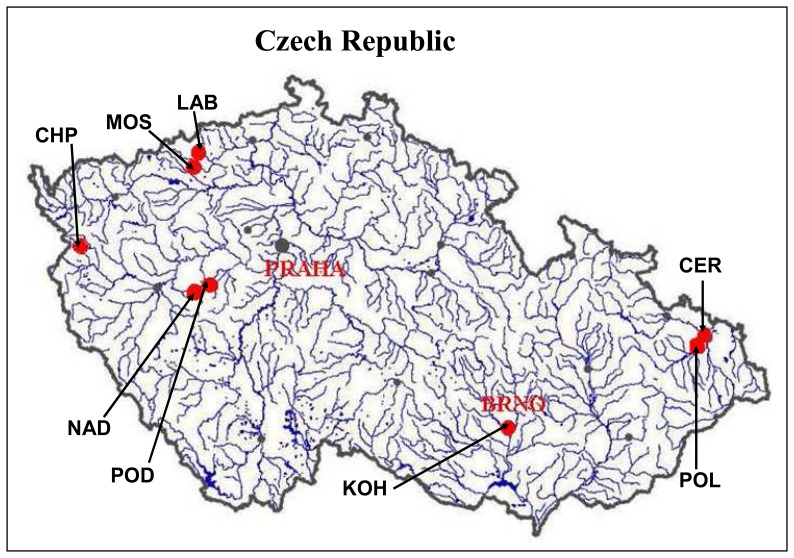
Map of sampling sites: CHP – Planenský potok (creek), MOS – Bílina: U Mostu, LAB – Bílina: U Labutí, NAD – Červený potok (creek), POD – Červený potok (creek), KOH – Kohoutovický potok (creek), POL – Polančice, CER – Černý potok (creek).

The larvae of freshwater midges *Chironomus riparius* were used in this study. The laboratory populations have been cultured according to standard procedures in siliceous sand overlaid by defined media at 20+-2°C, with constant humidity and 16 h light: 8 h dark photoperiod (US EPA, 2000). The samples for analysis of thiols were collected after fifteen days of exposure of chironomid larvae to sediment artificially contaminated by cadmium (Cd^2+^ ) at concentrations of 50 ng/g or 50 μg/g. The control samples were reared under same conditions without exposure to heavy metal. In the second part of the study, environmentally exposed chironomid larvae (3^rd^ instar) have been collected from eight field sites with various level of pollution by heavy metals. All the sampling sites were located at smaller streams with abundant chironomid populations. Several sites were in a close proximity of large industrial works, which are the potential sources of pollution including heavy metals (Fig. 1). The control samples were the same as in the first part of the study.

### 2.3 Heavy metals analysis

Heavy metals content in sediment samples was evaluated based on Aqua Regia leaching process and after total decomposition of silicate matrix. Inductively coupled plasma – mass spectrometry (ICP-MS) (Agilent 7500ce, Agilent Technologies, Japan) was used for determination of heavy metals in Aqua Regia leachate and total decomposed sediment samples. Elements (isotopes) suffering from polyatomic interferences were measured in He collision mode using Octopole Reaction System.

### 2.4 Preparation of biological samples for electrochemical analysis

Larvae of freshwater midges (app. 0.2 g) were homogenized using liquid nitrogen. The homogenate was quantitatively transferred to test tube and vortexed (Vortex Genie, USA) for 15 min at room temperature. The vortexed sample was prepared by heat treatment. Briefly, the sample was kept at 99 °C in a thermomixer (Eppendorf 5430, USA) for 15 min with occasional stirring, and then cooled to 4°C. The denatured homogenates were centrifuged at 4°C, 15 000 *g* for 30 min (Eppendorf 5402, USA). Heat treatment effectively denature and remove high molecular weight proteins from samples [10,12,53].

### 2.5 Stationary electrochemical analyser – Adsorptive transfer stripping differential pulse voltammetry Brdicka reaction – MT content

Electrochemical measurements were performed using an AUTOLAB analyser (EcoChemie, The Netherlands) connected to VA-Stand 663 (Metrohm, Switzerland), using a standard cell with three electrodes. The three-electrode system consisted of hanging mercury drop electrode as working electrode, an Ag/AgCl/3 M KCl reference electrode and a glassy carbon auxiliary electrode. For smoothing and baseline correction the software GPES 4.4 supplied by EcoChemie was employed. The Brdicka supporting electrolyte containing 1 mM Co(NH_3_)_6_Cl_3_ and 1 M ammonia buffer (NH_3_(aq) + NH_4_Cl, pH = 9.6) was used and changed after five measurements, surface-active agent was not added. AdTS DPV Brdicka reaction parameters were as follows: initial potential of −0.6 V, end potential −1.6 V, modulation time 0.057 s, time interval 0.2 s, step potential of 1.05 mV, modulation amplitude of 250 mV, E_ads_ = 0 V. Temperature of the supporting electrolyte was 4 °C. For other experimental conditions see in Ref. No. [10].

### 2.6 Stationary electrochemical analyser coupled with autosampler – Differential pulse voltammetry Brdicka reaction – Total content of thiols

Electrochemical measurements were performed with 747 VA Stand instrument connected to 746 VA Trace Analyzer and 695 Autosampler (Metrohm, Switzerland), using a standard cell with three electrodes and cooled sample holder (4 °C). A hanging mercury drop electrode (HMDE) with a drop area of 0.4 mm^2^ was the working electrode. An Ag/AgCl/3M KCl electrode was the reference and glassy carbon electrode was auxiliary electrode. The Brdicka supporting electrolyte mentioned in Section 2.5 was used and changed per one analysis. The DPV parameters were as follows: initial potential of −0.7 V, end potential of −1.75 V, modulation time 0.057 s, time interval 0.2 s, step potential 2 mV, modulation amplitude -250 mV, E_ads_ = 0 V. All experiments were carried out at temperature 4 °C (Julabo F12, Germany). For smoothing and baseline correction the software GPES 4.9 supplied by EcoChemie was employed.

**Figure 2.**
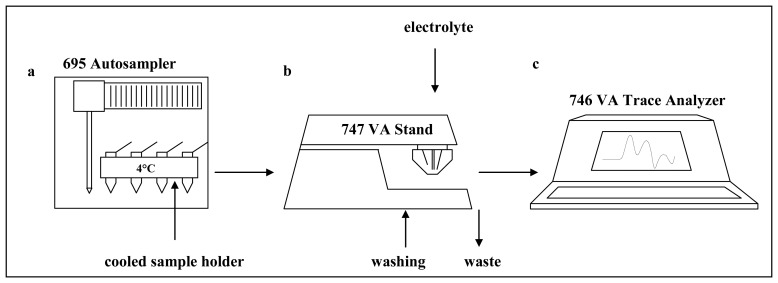
Scheme of stationary electrochemical analyser coupled with autosampler: (**a**) 695 Autosampler with cooled sample holder, (**b**) 747 VA Stand instrument with potentiostat/galvanostat using a standard cell with three electrodes and (**c**) 746 VA Trace Analyzer for data processing. Vessels with washing water and the supporting electrolyte are other parts of instrument.

A measurement proceeds as follows: A sample is positioned on the thermostatic sample holder (4 °C). The electrochemical cell is rinsed with distilled water (3 × 25 ml MiliQ water) using three computer controlled pumps. After draining of the water the supporting electrolyte (temperature 4 °C) is pipetted into the washed cell. Further a sample is introduced using the autosampler. The syringe from the autosampler is rinsed and the sample is injected to the cell. The measurement itself consists of a few following processes: At first, the injected sample is accumulated on the surface of hanging mercury drop electrode at open circuit for two minutes. At second, the current responses as function of various potentials are measured. At third, the measured values are processed by 746 VA Trace Analyser and transferred to a personal computer. At fourth, the transferred data is then processed using GPES 4.9 software (Fig. 2).

### 2.7 Statistical analyses

Data were processed using MICROSOFT EXCEL® (USA). Results are expressed as mean ± S.D. unless noted otherwise. Differences with p < 0.05 were considered significant (t-test was applied for means comparison).

## 3. Results and Discussion

Larvae of freshwater midges (*Chironomus riparius*) were used for monitoring of pollution of environment by heavy metals already more than 25 years ago [54]. Since then midges and mosquito larvae have been employed for assessment of environmental contamination [55-59]. As mentioned in “Introduction” section, level of MT determined in animal blood and tissues is thought to be a marker of heavy metal stress. Brdicka reaction is very promising analytical method to determine MT due to its very low detection limit [10], however, automated analyser is needed to analyse tens of real samples.

### 3.1 Stationary electrochemical analyser coupled with autosampler

**Figure 3.**
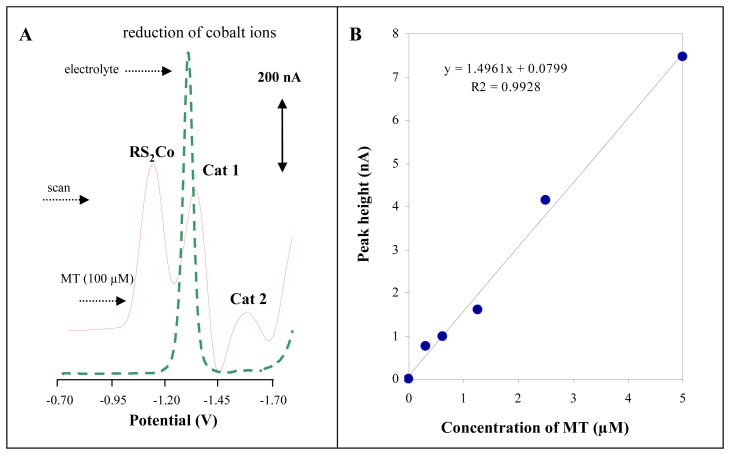
(**A**) Typical differential pulse voltammograms of the supporting electrolyte (dashed line) and MT (100 μM). (**B**) Dependence of Cat2 peak height on MT concentration.

Automatic autosampler injecting low sample volumes (units of μl) coupled with stationary electrochemical analyser was used to overcome a lack of using of Brdicka reaction to analyse tens even hundreds of real samples (Fig. 2). A measurement is carried out automatically under the control of microprocessor within five minutes. The typical voltammograms of the Brdicka supporting electrolyte and MT (100 μM) are shown in Fig. 3A. The calibration curve (dependence of Cat2 peak height on MT concentration, y = 1.4961 + 0.0799, R^2^ = 0.9928) obtained within the range from 0.25 to 5 μM is shown in Fig. 3B. Relative standard deviation of measurements was 3.6 %. The detection limit for MT estimated as 3 S/N was 5 nM. The detection limits (3 S/N) were calculated according to Long [60], whereas N was expressed as standard deviation of noise determined in the signal domain. The proposed methodology was utilized for determination of MT levels in larvae of freshwater midges exposed to cadmium(II) ions.

### 3.2 Determination of MT content in larvae of freshwater midges exposed to cadmium(II) ions

**Figure 4.**
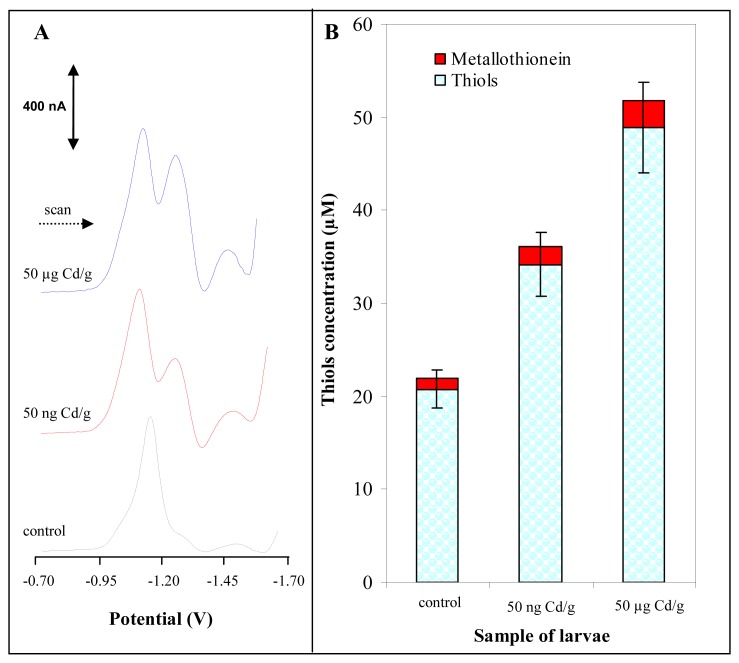
(**A**) Typical differential pulse voltammograms of samples obtained from larvae of freshwater midges exposed to 0 ng Cd/g, 50 ng Cd/g and 50 μg Cd/g. (**B**) The content of total thiols (stationary electrochemical analyser coupled with autosampler) and metallothionein (stationary electrochemical analyser) measured in the larvae exposed to cadmium(II) ions under controlled experimental conditions.

Martinez et al. published a paper on study of morphological deformities in larvae of insect from chironomidae family collected in heavy metal contaminated areas [61]. The authors also determined content of various heavy metals as As, Cd, Ni, Pb, Zn and Ni. They found a significant correlation between metal concentrations and deformity rates for all metals except Ni. To our knowledge a correlation between MT in Chironomids and heavy metals exposure has not been studied so far. In our first laboratory experiments, chironomid larvae have been exposed to sediment contaminated by cadmium(II) ions at concentrations of 50 ng/g or 50 μg/g for fifteen days. One may expected that correlation between MT content and concentration of the heavy metal could be assessed. The larvae exposed to cadmium doses showed no visible marks of heavy metal intoxication because they developed and behaved same as control group. At the very end of the exposure the larvae were killed, washed in distilled water and frozen (-20°C) prior to analysis. The samples of larvae were prepared according to procedure mentioned in “Material and Methods” section. The typical DP voltammograms of real samples are shown in Fig. 4A. The characteristic peaks obtained Cat1 and Cat2 were very well separated and developed. To quantify MT in the samples of interest the Cat2 signal was used. However, we have found previously that if we analysed real samples by the automatic electrochemical analyser (Fig. 2), we quantified not only MT content but also content of all heat stable low molecular mass thiols such as glutathione and others [62,63]. The content of the heat stable thiols in the treated larvae is shown in Fig. 4B. The total content of thiols was enhanced with cadmium(II) dose compared to control samples. Moreover, we attempted to determine MT itself by adsorptive transfer stripping differential pulse voltammetry Brdicka reaction (AdTS DPV Brdicka reaction) [10]. We found that the content of MT also increased with increasing dose of the heavy metal ions but more substantially. MT content in control samples was 1.2 μM, in larvae exposed to 50 ng Cd/g it was 2.0 μM and in larvae exposed to 50 μg Cd/g 2.9 μM. Based on the results obtained it follows that the automated analyser is suitable for routine determination of thiols content in larvae exposed to heavy metals and, thus, to assess heavy metal pollution of environment.

### 3.3 Determination of the content of heavy metals and thiols in chironomid larvae from the field study

The field study with sampling of chironomids populations from the environment has been conducted to examine the levels of studied thiols and MT in the wild populations. Chironomid larvae as well as sediment samples have been collected from eight field sites with different levels of pollution by heavy metals called LAB, KOH, CER, CHP, NAD, POD, MOS and POL. Most of these sites have been impacted by undergoing or former industrial activities, thus increased concentration of pollutants including heavy metals can be expected. The metals content in sediment extracts were determined by ICP-MS. We determined content of chromium, nickel, copper, zinc, arsenic, molybdenum, cadmium, tin and lead (Tab. 1). Using the simplified criterion for assessment of environmental contamination (sum of concentration of all heavy metals determined in the locality) we evaluated the localities with highest and lowest contamination. The most polluted locality was LAB followed by KOH, CER, CHP, NAD, POD, MOS and POL. Besides the sum of the heavy metal concentrations sediments samples from the LAB locality contained the highest concentrations of seven out of the ten analyzed metals, particularly cobalt, nickel, copper, zinc, arsenic, cadmium and tin.

**Figure 5.**
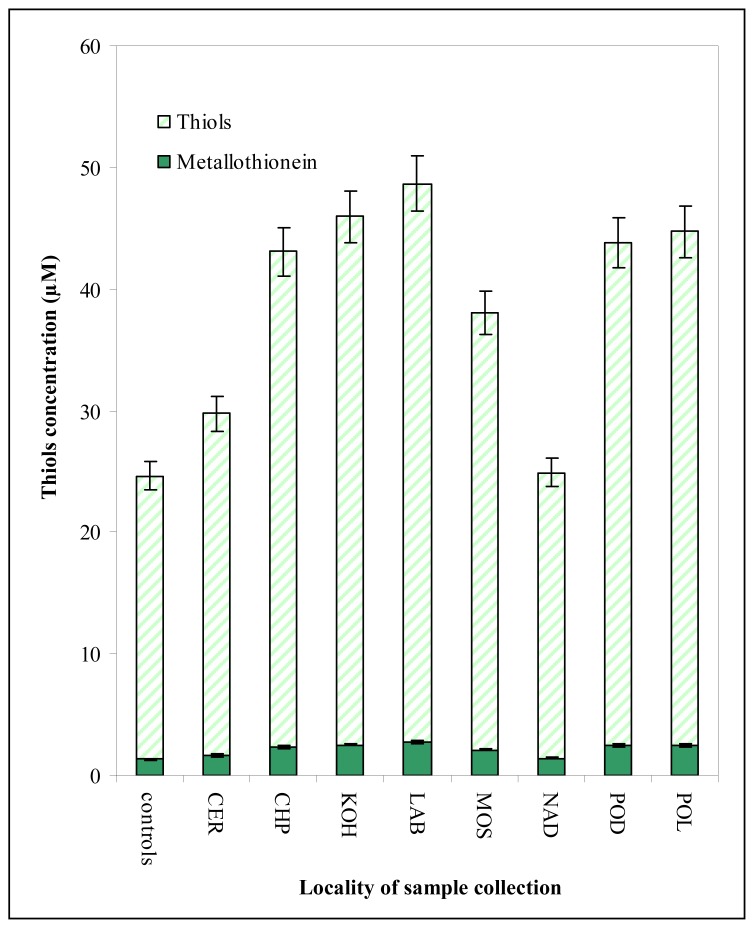
The content of total thiols and metallothionein measured in the larvae sampled from eight field sites with various level of pollution by heavy metals.

Moreover total content of thiols and MT in the chironomid larvae (3^rd^ instar) sampled at the same sites as sediments was determined electrochemically (Fig. 5). The thiols and MT content determined in the larvae sampled was higher in comparison to control ones (from 102 to 198 % of content in control group). However, the content of the target molecules in the larvae samples at CER and NAD localities was lower about 30-40 % compared to other field localities. This difference could be associated with many factors hardly identified due to natural origin of the samples. After exclusion of results obtained from these two localities (CER and NAD) due to the lowest levels of MT, the good correlation (y(*sum of content of heavy metals as mg/kg*) = 0.0004×(content of MT, μM) + 2.0917; R^2^ = 0.8294) between metallothionein content and observed contamination expressed as total heavy metal concentration was obtained.

## 4. Conclusion

We have shown that the level of thiols in larvae of freshwater midges can be determined by using stationary electrochemical analyser coupled with autosampler. Moreover, we have compared the total thiols content and MT level determined by AdTS DPV Brdicka reaction and found that these values well correlated.

## Figures and Tables

**Table 1.** Content of heavy metals in the sediments from the studied field sites.

**Locality**	Heavy metals (mg/kg)*										
	Cr	Co	Ni	Cu	Zn	As	Mo	Cd	Sn	Pb	***Sum***
MOS	23.7	28.9	44.0	32.0	153	33.8	1.5	0.7	0.8	23.9	***342.3***
LAB	74.4	170*	249*	346*	715*	81.7*	17.9	2.8*	5.8*	74.0	***1736.6***
CER	243*	21.0	38.7	114	417	9.6	8.1	0.9	1.5	152	***1005.8***
POL	17.3	4.9	14.8	23.9	132	3.9	0.6	0.3	0.5	15.4	***213.6***
NAD	57.0	24.3	33.6	38.3	336	10.2	118	1.3	3.8	39.2	***661.7***
POD	51.1	20.3	41.6	46.9	264	19.2	133*	2.5	3.4	41.6	***623.6***
CHP	66.6	10.0	39.9	43.1	567	7.6	3.3	0.6	1.0	22.7	***761.8***
KOH	121	23.3	41.7	93.1	667	5.9	4.0	0.6	2.2	86.9*	***1045.7***

* … The highest concentration of the metal.
